# Prenatal androgen induced lean PCOS impairs mitochondria and mRNA profiles in oocytes

**DOI:** 10.1530/EC-19-0553

**Published:** 2020-01-25

**Authors:** Neil R Chappell, Beth Zhou, Amy K Schutt, William E Gibbons, Chellakkan S Blesson

**Affiliations:** 1 Reproductive Endocrinology and Infertility Division, Department of Obstetrics and Gynecology, Baylor College of Medicine and Family Fertility Center, Texas Children’s Hospital, Houston, Texas, USA

**Keywords:** PCOS, mitochondria, oocytes, glucose intolerance

## Abstract

Polycystic ovary syndrome (PCOS) is the most common ovulatory defect in women. Although most PCOS patients are obese, a subset of PCOS women are lean but show similar risks for adverse fertility outcomes. A lean PCOS mouse model was created using prenatal androgen administration. This developmentally programmed mouse model was used for this study. Our objective was to investigate if mitochondrial structure and functions were compromised in oocytes obtained from lean PCOS mouse. The lean PCOS mouse model was validated by performing glucose tolerance test, HbA1c levels, body weight and estrous cycle analyses. Oocytes were isolated and were used to investigate inner mitochondrial membrane potential, oxidative stress, lipid peroxidation, ATP production, mtDNA copy number, transcript abundance and electron microscopy. Our results demonstrate that lean PCOS mice have similar weight to that of the controls but exhibit glucose intolerance and hyperinsulinemia along with dysregulated estrus cycle. Analysis of their oocytes show impaired inner mitochondrial membrane function, elevated reactive oxygen species (ROS) and increased RNA transcript abundance. Electron microscopy of the oocytes showed impaired mitochondrial ultrastructure. In conclusion, the lean PCOS mouse model shows a decreased oocyte quality related to impaired mitochondrial ultrastructure and function.

## Introduction

Polycystic ovary syndrome (PCOS) is the most common ovulatory disorder in the world, affecting 5–10% of women, translating to ~100 million women worldwide (1). PCOS is associated with increased obstetric risks including preeclampsia, gestational diabetes, preterm delivery and higher infant mortality (2). PCOS patients have higher miscarriage rates (3, 4), higher risk of ovarian hyperstimulation (OHSS) and increased risks in offspring born after assisted reproduction technology (ART) (5). Further, there are longstanding implications in chronic disease states such as hypertension, diabetes, depression, stroke and some cancers (2).

PCOS is a broad and complex disease comprised of several aberrations in physiology, leading to variable phenotypes. Diagnosis of PCOS is based on the Rotterdam criteria: polycystic ovarian morphology, irregular menses and clinical or laboratory evidence of hyperandrogenemia (6). At least two of these three criteria will qualify a patient for the diagnosis of PCOS, provided routine workup excludes other etiologies (6). Up to 80% of PCOS patients are obese, and obesity is associated with a wide range of adverse events (7, 8, 9), many of which are closely associated with outcomes seen in PCOS literature (10, 11). This makes obesity the quintessential confounder in the study of PCOS. Recently, a subpopulation of PCOS patients, that is, not obese, coined ‘lean PCOS’, has elicited interest among researchers (12, 13, 14, 15, 16, 17). This group comprises 20–30% of the PCOS population and provides the opportunity to look at PCOS independent of obesity.

Mouse models have long served as a useful platform to study PCOS, and several models have been described, including a ‘lean PCOS’ mouse model via administration of androgens prenatally during the critical window of fetal oogenesis (18, 19, 20, 21, 22, 23). This model has since been well characterized metabolically and exhibits a PCOS-like phenotype with hyperandrogenemia, increased LH activity, irregular cyclicity and abnormal glucose/insulin tolerance while maintaining the same weight and BMI as the control population (18, 20, 21). Despite the strong foundational data describing the metabolic components of this model, research on the reproductive system of these animals, in particular the oocyte itself, remains sparse. Prior studies have shown impaired mitochondrial function with hyperandrogenic environments in the oocyte to have negative effects on reproductive outcomes (24, 25, 26, 27, 28, 29). PCOS women by their nature are hyperandrogenic and thus may undergo these alterations (30, 31, 32). Our hypothesis is that prenatal androgen administration results in a lean PCOS phenotype with impaired mitochondrial structure and function in the oocytes.

## Materials and methods

All experiments were approved through the Institutional Animal Care and Use Committee of Baylor College of Medicine (AN-7156). To create the lean PCOS mouse model, 8-week-old C57/Bl6 female mice (*n* = 26) were mated with males of proven fertility. Copulatory plugs were visualized to confirm mating on the following morning, which was considered day 0.5. Pregnant dams were injected with 250 µg of dihydrotestosterone (DHT, Sigma-Aldrich) prepared in sesame oil (Texas Lab Supply, Lubbock, TX, USA) or vehicle only on 16.5, 17.5 and 18.5 days post coitus. All male pups were culled at weaning, and female pups (*n* = 30 controls and *n* = 33 DHT) were used for the experiments. One pup each from each dam was used for each experiment and sisters were not used in the same experiments. From within each litter, pups were randomly chosen for different experiments.

Pups were weighed and measured from snout to anus in millimeters at 3, 4, 8, and 12 weeks up to killing at 16 weeks to calculate their BMI. Test for estrous cyclicity was performed at 3 months of age by observing the vaginal cytology using vaginal smears as previously described (33, 34, 35) for approximately 21 days or four consecutive cycles. Glucose tolerance test (GTT) was performed on 12-week-old mice as in previously published mouse models (21, 36). Hemoglobin A1c levels were analyzed at the time of killing at 16 weeks using the A1cNow+ test kit (PTS Diagnostics, Indianapolis, IN, USA) following the manufacturer’s instructions. Due to the sub-fertile nature of the animal model, in order to study the oocyte, it was necessary to perform superovulation to retrieve oocytes. The protocol is detailed in the Supplementary Materials (see section on supplementary materials given at the end of this article).

Mitochondrial function was evaluated on oocytes by single cell imaging experiments using fluorescent probes and the images were analyzed using Image J software. Inner mitochondrial membrane (IMM) potential was measured using JC-1 dye (Affymetrix), reactive oxygen species (ROS) formation was measured using CellRox Green (ThermoFisher) and lipid peroxidation was measured by using BODIPY (Life Technologies). Single cell imaging of oocytes for IMM, ROS and lipid peroxidation were performed using two to five oocytes/study/group from five to eight mice.

ATP concentration was measured pooling 5 to 15 oocytes/mouse, using a luciferase assay kit (ThermoFisher). RNA transcript abundance was measured using qPCR. RNA was isolated and cDNA was amplified using three to five oocytes collected from each mouse. Genes were amplified using specific primers using cDNA library as templates. Genomic DNA was isolated and amplified to measure mitochondrial DNA copy number. During killing, ovaries from 4-month-old unstimulated mice in diestrus were collected and processed for transmission electron microscopy (TEM). The ‘*n*’ in each study corresponds to the number of mice used in each experiment. Detailed protocols utilized are described in the Supplementary Materials. Student’s *t*-test, chi square and ANOVA were used where appropriate for all measurements. *P* < 0.05 was considered statistically significant. The data were analyzed using GraphPad Prism version 6.0.

## Results

### Control and PCOS mouse showed similar body weights and BMI

**Figure 1:**
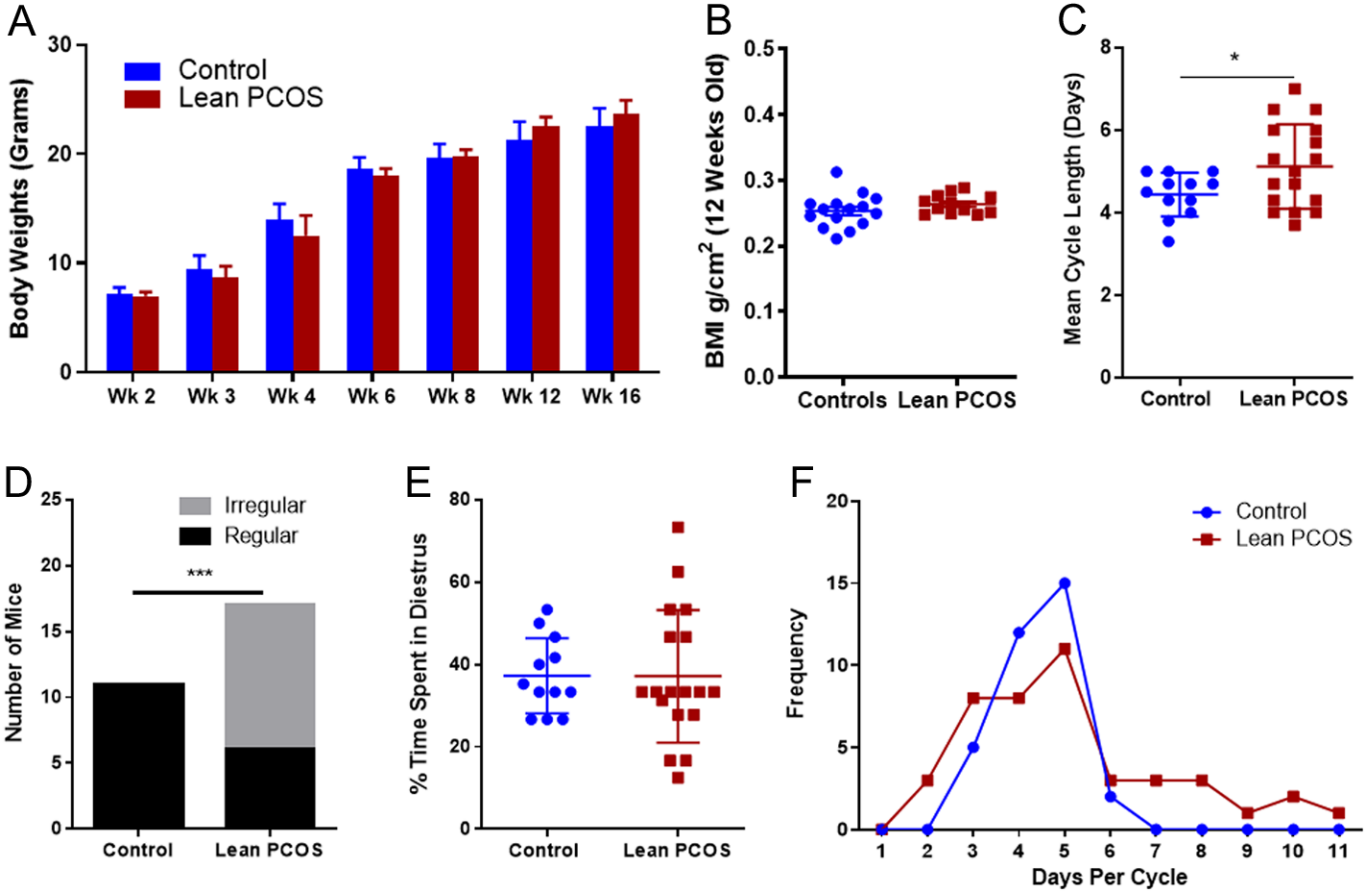
Lean PCOS mice displayed similar body weight (A) and BMI (B) when compared to controls. They also showed irregular cycles when compared to controls. The mean cycle length (C) in the lean PCOS group was longer, and more mice exhibited irregular cycles (D) and had more variation in the overall time spent in diestrus (E), with a wider variation in the days spent per estrus cycle (F). (*n* = 11–15 in controls and 18–19 in lean PCOS, *P < 0.05 and ***P < 0.001).

The lean PCOS mouse model exhibited similar weights when compared to controls throughout the experimental period (Fig. 1A). They did not show any difference at 16 weeks at the time of killing (control 22.48 ± 0.47 g vs lean PCOS 23.67 ± 0.29 g at 16 weeks, *P* = 0.19). Further, there was no difference in BMI at 12 weeks between the two groups (control 0.25 ± 0.007 g/cm^2^ vs lean PCOS 0.26 ± 0.004 g/cm^2^, *P* = 0.22 Fig. 1B).

### Lean PCOS mice had irregular cycles

Lean PCOS mice displayed irregular cycles when compared to controls. The mean cycle length for the lean PCOS mice was longer than that of the controls, indicating longer estrus cycles (control 4.44 ± 0.15 days, *n* = 12, vs lean PCOS 5.12 ± 0.25 days, *P* < 0.05; Fig. 1C). All control mice had regular estrus cycles (defined as 3–5 days per cycle); however, a significant proportion of lean PCOS mice had did not have regular cycles (control 100% regular cycles vs lean PCOS 35% regular cycles, *n* = 17, *P* < 0.001; Fig. 1D). The percentage of time of spent in the diestrus was calculated for each mouse (days in diestrus/number of days monitored). For the control population, mice spent a similar time in diestrus with a small variation (37.2% ± 2.6), while lean PCOS mice showed significant variations (37.1% ± 3.8) (Fig. 1E) with days ranging from 2–10 as opposed to 3–5 days in controls (Fig. 1F). Serum obtained at 4 months was used to measure anti-Mullerian hormone (AMH) levels using standard ELISA kits, and no differences were noted between the control group and the lean PCOS group (control 68.4 ± 7.74 ng/mL vs lean PCOS 55.08 ± 7.8 ng/mL for AMH).

### Lean PCOS mice were glucose intolerance but normal hemoglobin A1c levels

**Figure 2:**
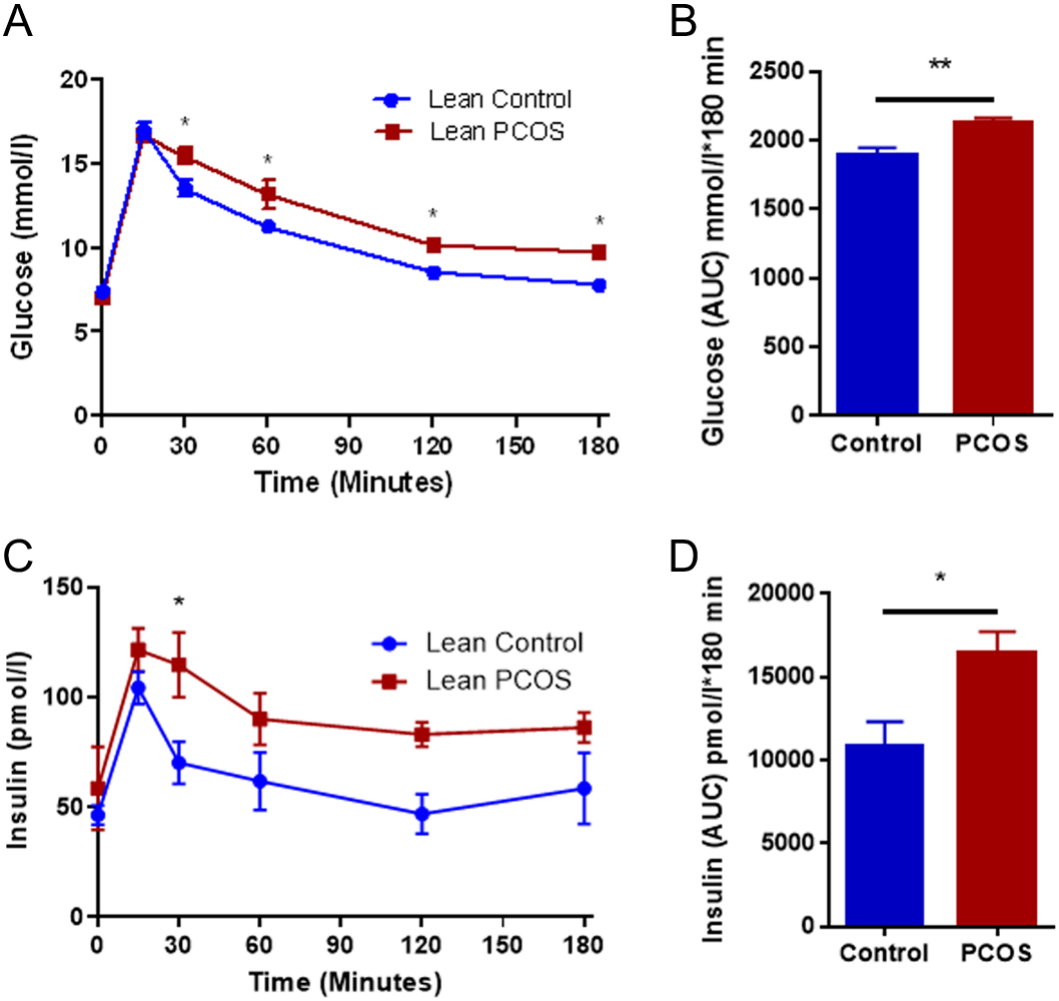
The lean PCOS mouse model displayed glucose intolerance (A) with increased glucose area under the curve (AUC) (B). Additionally, the lean PCOS mouse model also demonstrated hyperinsulinemia (C) with elevated insulin AUC (D). (*n* = 7–8 in controls and *n* = 8 lean PCOS, *P < 0.05 and **P < 0.01).

Glucose values were consistently higher at 30 min (control 13.5 ± 0.5 mmol/L vs lean PCOS 15.5 ± 0.5 mmol/L, *P* < 0.01), 60 min (control 11.3 ± 0.3 mmol/L vs lean PCOS 13.2 ± 0.9 mmol/L, *P* < 0.01), 120 min (control 8.5 ± 0.3 mmol/L vs lean PCOS 10.2 ± 0.2 mmol/L, *P* < 0.01) and 180 min (control 7.8 ± 0.2 mmol/L vs lean PCOS 9.7 ± 0.1 mmol/L, *P* < 0.01) during GTT (Fig. 2A). Interestingly, fasting glucose did not show any difference between the groups (Fig. 2A). The lean PCOS mice exhibited glucose intolerance when compared to controls with an increase in the GTT area under the curve (AUC), demonstrating an overall increase in glucose intolerance in the lean PCOS group when compared to the controls (Fig. 2B, control 1891 ± 53.81 mmol/L × 180 min vs lean PCOS 2121 ± 39.56 mmol/L × 180 min, *P* < 0.01). The lean PCOS group showed evidence of hyperinsulinemia at the 30 min (Fig. 2C, control 70.134 ± 9.68 pmol/L vs lean PCOS 114.7 ± 14.71 pmol/L, *P* < 0.05), with a higher AUC compared to controls (Fig. 2D, control 10,843 ± 1484 pmol/L × 180 min vs lean PCOS 16,462 ± 1261 pmol/L × 180 min, *P* < 0.05). However, there were no differences in the insulin levels in any other time point during the GTT. Further, there was no difference in hemoglobin A1c between the lean PCOS group and controls (control 4.3 ± 0.12% vs lean PCOS 4.46 ± 0.11%).

### Inner mitochondrial membrane potential was compromised in lean PCOS oocytes

**Figure 3:**
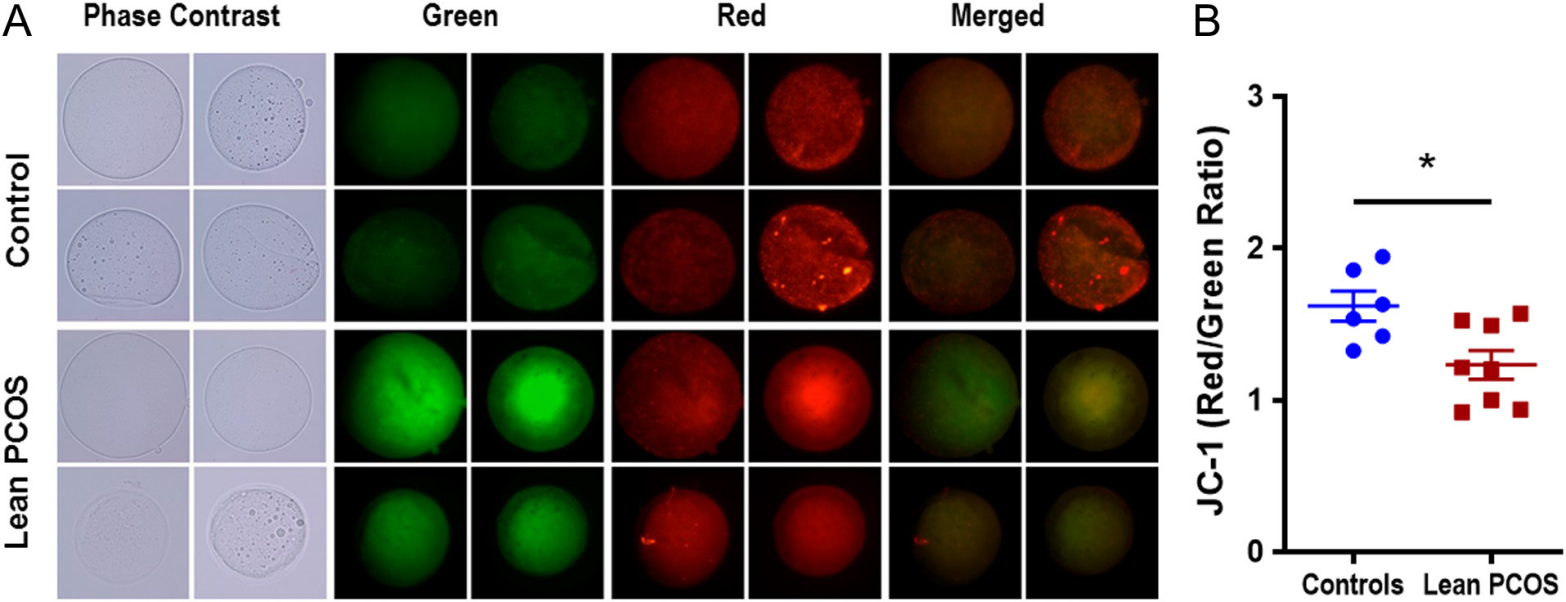
Pictorial representation (A) of control (upper panel) and lean PCOS (lower panel) model. Phase contrast microscopy, followed by green fluorescence, red fluorescence and merged fluorescent images using JC-1 dye at 60× magnification. The lean PCOS mice show a lower red to green ratio compared to controls (B) indicating compromised mitochondrial function. (*n* = 5 in controls and *n* = 8 in lean PCOS, *P < 0.05).

Changes in IMM potential were assessed in live oocytes obtained from controls and lean PCOS mouse using JC-1, a dye that fluoresces red when the inner mitochondrial membrane is charged and green when depolarized. JC-1 is a marker for overall mitochondrial health and function, as this membrane potential is essential for the electron transport chain to be able to produce ATP (29, 37). Our analysis showed that the lean model exhibited a lower red to green ratio in oocytes collected, indicating a lower IMM potential (control 1.62 ± 0.1 vs lean PCOS 1.23 ± 0.09, *P* < 0.05; Fig. 3A and B).

### Oocytes from lean PCOS mice exhibited increased ROS

**Figure 4:**
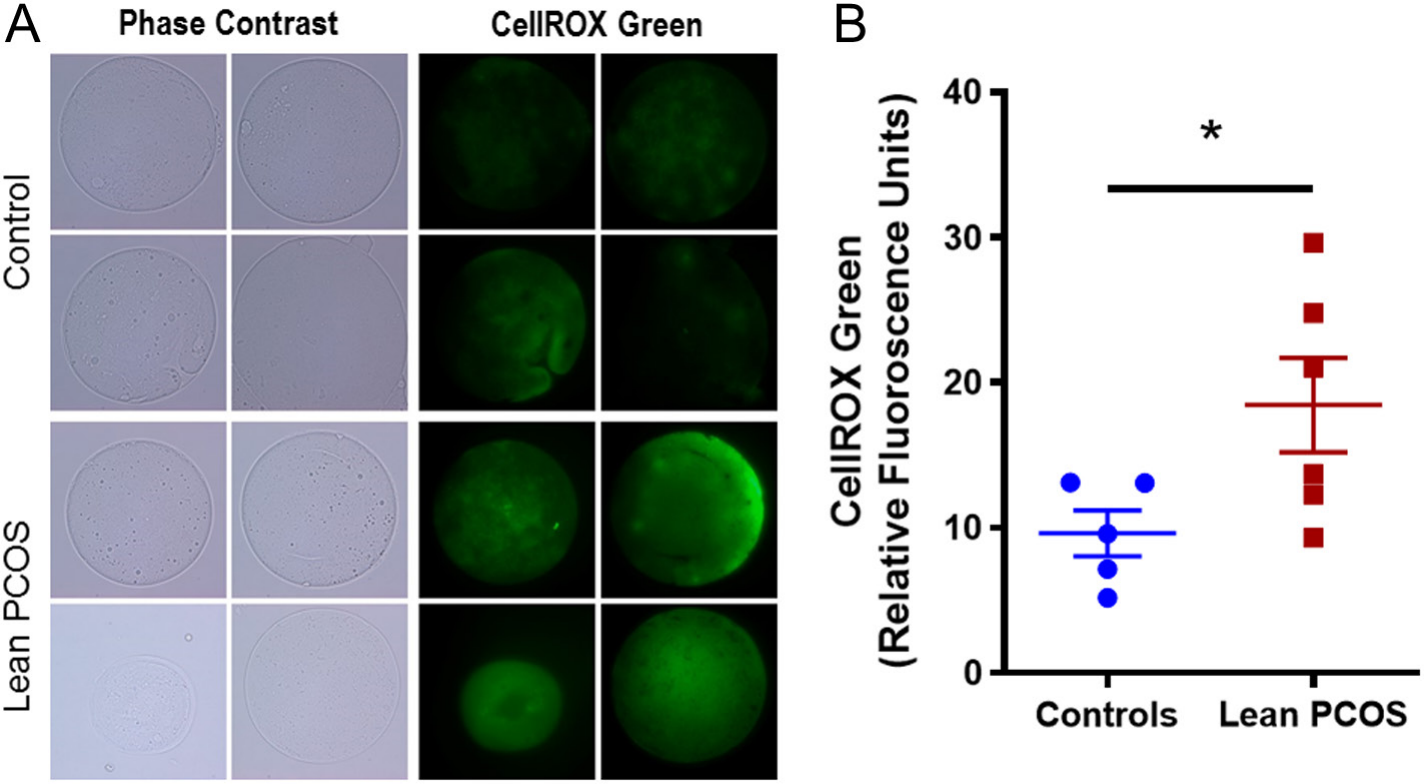
Pictorial representation (A) of control (upper panel) and lean PCOS (lower panel) model. Phase contrast microscopy, followed by green fluorescence using CellRox Green dye at 60× magnification. The lean PCOS mice have a higher mean RFU value indicating presence of higher reactive oxygen species concentration when compared to controls (B). (*n* = 5 in controls and *n* = 6 in lean PCOS, *P < 0.05).

ROS was measured using CellRox Green which fluoresces when oxidized by reactive oxygen species (ROS), and thus the higher the fluorescent signal measured in relative fluorescent units (RFUs), the higher the amount of ROS present. In the lean PCOS model, we observed higher RFUs compared to controls indicating a higher concentration of ROS (control 9.62 ± 1.58 RFUs vs lean PCOS 18.46 ± 3.25 RFUs, *P* < 0.05; Fig. 4A and B).

### Lipid peroxidation was similar between the control and PCOS oocytes

**Figure 5:**
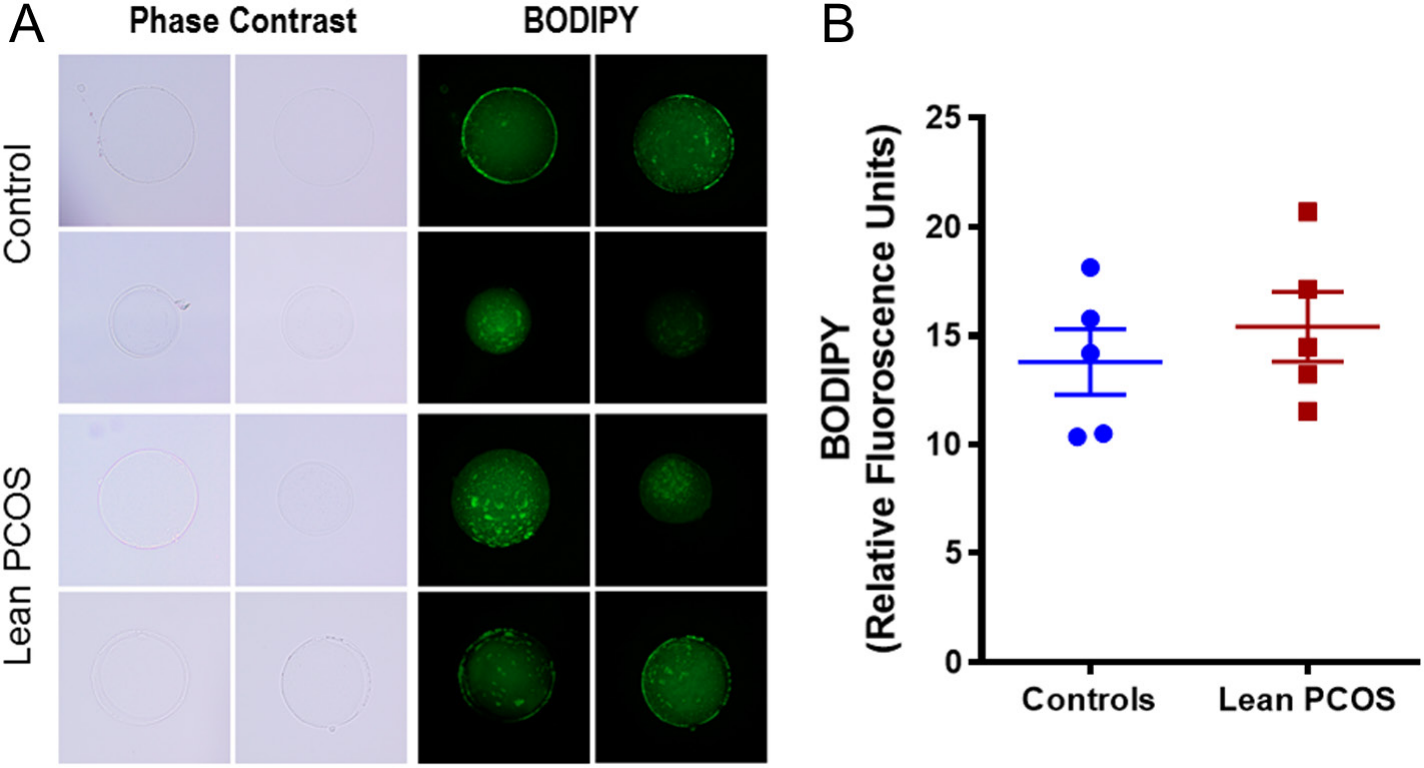
Pictorial representation (A) of control (upper panel) and lean PCOS (lower panel) model. Phase contrast microscopy, followed by green fluorescence using BODIPY dye at 60× magnification. The lean PCOS mice have a similar mean RFU value when compared to controls indicating no differences in lipid peroxidation between the groups (B). (*n* = 5 in controls and *n* = 5 in lean PCOS).

Lipid peroxidation was measured using BODIPY, a dye used to measure lipid peroxidation. Mitochondria are a major site of lipid metabolism, and peroxidation of lipids in mitochondria has been used as a marker for mitochondrial function (25). We measured mitochondrial lipid peroxidation in oocytes using a fluorescent dye, BODIPY. We observed no differences between the control and lean PCOS oocytes (control 13.8 ± 1.51 RFUs vs lean PCOS 15.41 ± 1.61 RFUs; Fig. 5A and B).

### mtDNA copy number and ATP levels showed no differences

Mitochondrial DNA copy number and ATP production were assessed in oocytes obtained from control and lean PCOS groups. Mitochondrial DNA copy number was assessed by qPCR of a mitochondrial gene (mtCo1) compared with a somatic reference gene (tubulin). Our data show that there were no difference noted in mtDNA copy number between the groups (control 1127 ± 707 vs lean PCOS 1161 ± 572). ATP contents were measured from oocyte lysates and there was no difference between the groups (control 21.08 ± 4.26 vs lean PCOS 22.15 ± 4.66).

### Lean PCOS oocytes showed increased transcript abundance

**Figure 6:**
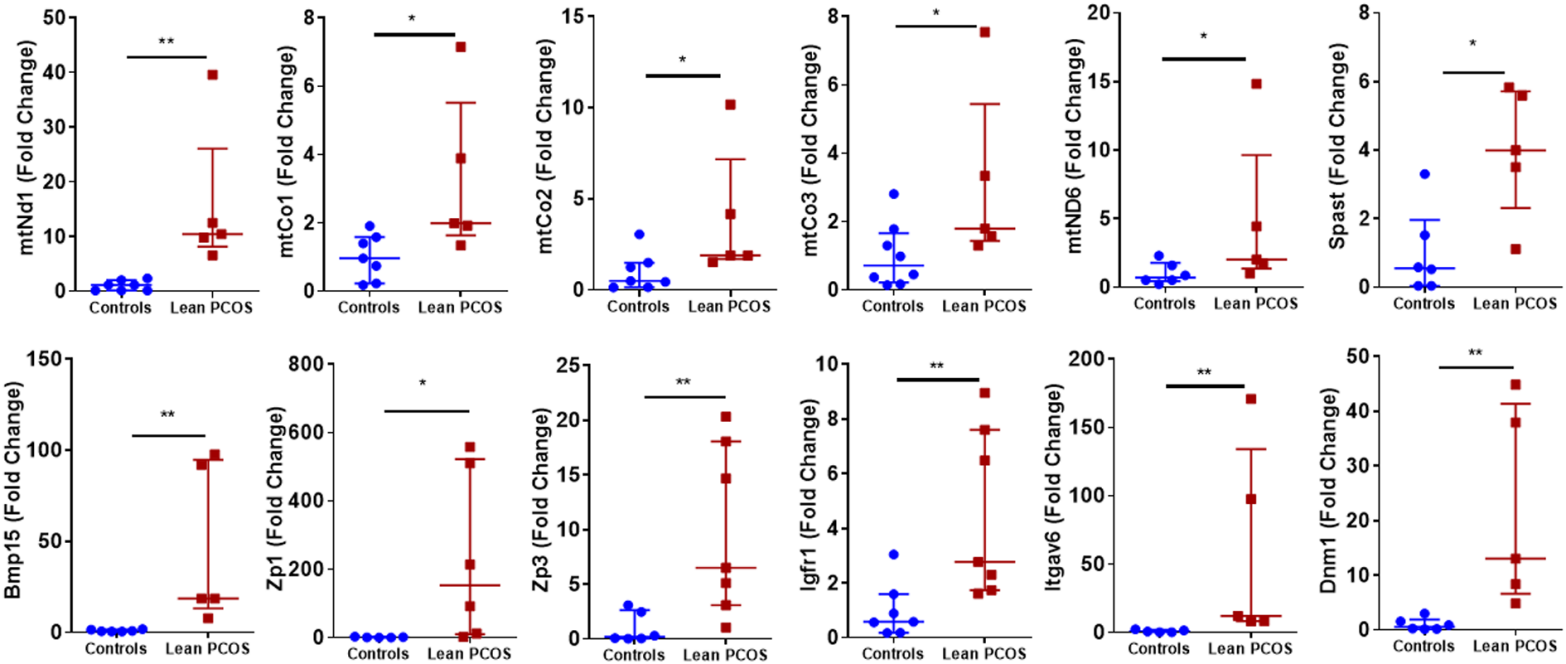
RNA transcript abundance in the control group vs lean PCOS mouse model. (*n* = 5–8, *P < 0.05, **P < 0.01).

RNA transcript abundance was quantified using qPCR for nuclear and mitochondrial genes. Interestingly all the statistically significant differences showed increased abundance in lean PCOS group (Fig. 6). qPCR was performed for 39 genes known to be involved in PCOS and reproduction. Our results showed that five nuclear genes and seven mitochondrial genes were upregulated. Of the nuclear genes that were investigated, there was a significant increase (*P* < 0.05) in gene transcripts involved in centrosome formation (Spast), folliculogenesis (Bmp15, Zp1, Zp3 and Igfr1) and cell adhesion (Itgav6 and Dnm1). Mitochondrial genes that were upregulated (*P* < 0.05) included genes involved with complex 1 of the electron transport chain (mtNd1 and mtNd6) and complex IV (mtCo1, mtCo2 and mtCo3). The mitochondrial genes mtNd2, mtNd3, mtNd4, mtCytb, mtAtp8 and mtRnr2 in addition to the nuclear genes Opa1, Mfn2, Ect2, Atrx, Cep70, Tacc1, Pcm1, Gdf9, Nobox, Zmic1, Kat2b, Ppp2r1a and Ppp2ca all showed a tendency to be more abundant; however, did not reach statistical significance. Mitochondrial genes mtNd4l, mtNd5, mtAtp6 and mtRnr1 as well as nuclear genes Gja1 and Sfrp4 showed similar expression in both controls and lean PCOS groups, and Xrcc1 showed a trend in lower abundance that did not reach statistical significance (Data not shown). A complete list of primers used and their details are given in the Supplementary Materials.

### Lean PCOS oocytes showed compromised mitochondrial ultrastructure

**Figure 7:**
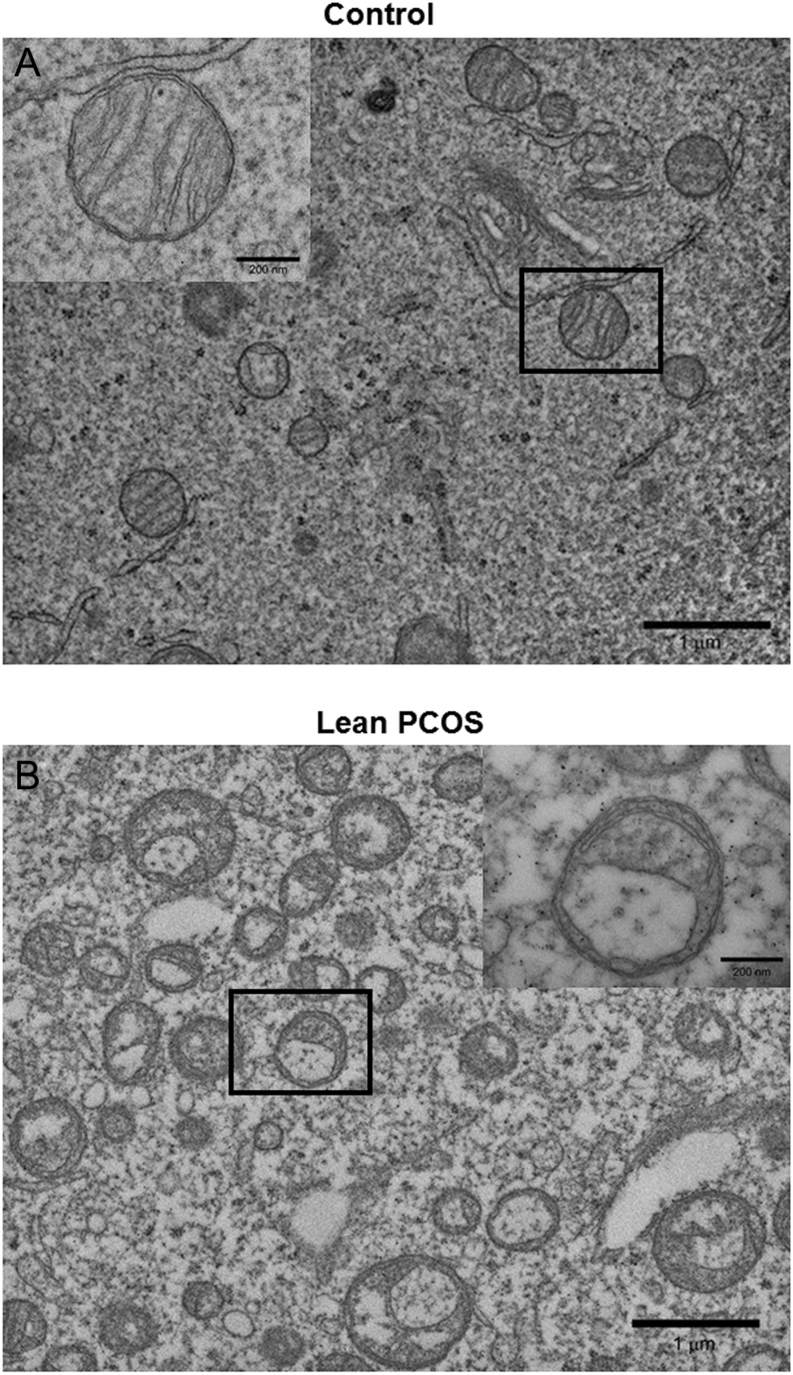
Representative transmission electron microscopy imaging of control (A) and lean PCOS (B) mouse oocyte mitochondria. Oocytes from lean PCOS mouse show abnormal mitochondrial structure with swollen cristae (*n* = 4 in each group).

TEM images of mouse oocytes showed that mitochondria in the lean PCOS group were structurally abnormal when compared to the controls. The controls had well rounded mitochondria with cristae at equal intervals. Further, they also had normal looking electron dense materials inside the mitochondria. Almost all the mitochondria observed had uniform shape with normal ultrastructure. Interestingly, almost all the mitochondria in oocytes from lean PCOS group showed aberrant ultrastructure with swollen cristae. Further, they were also severely vacuolated without any electron dense contents (Fig. 7A and B).

## Discussion

The lean PCOS mouse model demonstrated characteristics consistent with lean PCOS patients seen in clinical practice, with metabolic abnormalities, irregular cyclicity and evidence of ovulatory dysfunction with no increase in body weight or BMI. Although we noted a slight increase (1.2 g, ~5%) in the body weight of lean PCOS mice at 4 months, it did not reach statistical significance (*P* = 0.19). This increase may have some possible impact, despite the lack of statistical difference. Lean PCOS mice showed glucose intolerance and hyperinsulinemia during the GTT, suggesting compromised glucose metabolism as seen in the majority of lean PCOS patients (2, 38). Interestingly, there was no difference in hemoglobin A1c levels compared to controls, indicating a lack of persistent hyperglycemia in these mice. Rather, they showed compensatory hyperinsulinemia indicating that higher insulin secretion was able to maintain glucose homeostasis. Such compensatory increase in insulin levels are often observed in patients with metabolic diseases; however, if unchecked will ultimately progress to worsening insulin resistance and lead to pancreatic failure (39). Earlier studies have shown that this model has elevated testosterone levels and impaired estrogen feedback (18).

To the best of our knowledge, our study presents evidence for the first time that prenatal androgen administration affects oocyte mitochondrial structure and function. The critical window of oocyte development in a mouse begins around day 16–18 of *in utero* fetal development (the period which the DHT was administered in this model) and continues into neonatal life. Importantly, administration of DHT after this decisive window does not carry the same effects (40). In contrast, our study clearly shows that administration of DHT during this window of fetal development affects the mitochondrial structure and function of oocytes during adulthood. The oocyte is the largest cell in the body and mitochondria are abundantly present within oocytes (27). A primordial oocyte will only have a handful of mitochondria though, throughout folliculogenesis, that number will increase to over 100,000 (27). After ovulation, this abruptly ceases, and there will be no changes in mitochondrial abundance until blastulation (27, 41). Therefore, a healthy amount of functional oocyte mitochondria is essential in the formation and maintenance of a strong energy reserve for the early processes of embryogenesis from fertilization through the first cell divisions (42). Thus, the implications of impaired mitochondrial function at the level of the oocyte are clear from our findings and may help to explain the adverse outcomes seen in the PCOS population.

One of the main functions of mitochondria is to generate energy in the form of ATP, which is done via the electron transport chain (ETC) (43). The ETC is responsible for pumping protons across the IMM to generate a transmembrane gradient which is then utilized by an ATPase enzyme to create ATP (37). As the membrane potential across the IMM decreases, the ETC’s function is compromised, and this has been shown to be a marker for poor mitochondrial function (37). Further, mitochondria are also integral in reactive oxygen species (ROS) formation and scavenging and regulation of apoptotic pathways (44). While a certain amount of ROS is considered normal in the oocyte, increased ROS is linked to poor reproductive outcomes via developmental arrest, apoptosis and physical DNA damage (29, 43, 45, 46). Oocytes obtained from lean PCOS mice displayed compromised IMM potentials and increased ROS formation. Formation of ROS is tightly coupled to mitochondria; in fact, the mitochondrial genome is more susceptible to DNA damage from ROS than the nuclear genome (47). This is thought to act as a buffering mechanism, whereby excessive amounts of ROS can result in mitochondrial DNA damage which triggers apoptotic pathways before nuclear damage can accumulate to a dangerous degree (47). Mechanisms by which the mitochondria control ROS formation have been postulated to involve scavenger activity, increased antioxidant production and alterations in fission/fusion balance of mitochondrial number (43, 47, 48).

The lack of difference in mtDNA copy number and ATP concentration despite mitochondrial damage may be due to compensatory mechanisms in PCOS oocytes. Our recent study in androgen programmed neonatal ovaries showed similar mitochondrial damage possibly due to increased oxidative stress with increased basal ATP production (49). It is possible that persistent exposure to androgens later in life may trigger alterations in mitochondrial replication that could result in differences in ATP concentration or mitochondrial copy number.

The persistence of the RNA transcripts seen in the lean PCOS mouse has exciting implications. Unlike most cells whose mRNA transcripts lasts for only a few hours (50), an oocyte transcribes mRNAs to last several days (50) from fertilization to blastulation and implantation (50). The longevity of these mRNA transcripts in this unique environment is thought to be due to changes in the adenylation of the 3′ tail of the mRNA, increased expression of binding proteins, inhibition of degradation pathways and through the influence of miRNAs (50). Therefore, studies of RNA expression that would typically quantify gene expression in other cell lines are more appropriately considered to be measuring quantities of RNA transcripts created prior to ovulation in the case of the oocyte (50). The majority of the genes tested in this lean model showed increased abundance of RNA transcripts in oocytes obtained from lean PCOS group, suggesting a global difference in either transcription or stabilization of these transcripts (50). The mechanism for global increase in RNA transcript DHT exposed oocytes is not known. The stabilization and prolongation of mRNA half-life can be achieved by various mechanisms (50) or there may be altered expression of miRNA transcripts, which have been reported in PCOS cohorts (51). And finally, there may be increased storage of mRNA transcripts in PCOS. In fact, studies have shown increased expression of the gene GATA6 in PCOS, which is involved in mRNA storage in the oocyte (52).

KEGG analysis of the regulated genes indicate that oxidative phosphorylation (especially in Complex I and Complex IV of the ETC) is the major pathway involved in the dysregulation. One of the main functions of the mitochondria is energy generation via the ETC, and during folliculogenesis, the number of mitochondria increase exponentially. Therefore, decreased mRNA transcript abundance of proteins involved in the ETC may indicate compromised ETC function in these oocytes. Precisely how the programming affects the growth and maturation of the oocytes and embryos that may arise from these oocytes if fertilized is not yet understood. Although the mechanisms remain unknown, a prior study in neuronal tissue of mice exposed to androgen (DHEA) has shown impairment in Complex I of mitochondrial electron transport chain (26). Other pathways involved included ovarian steroidogenesis, meiosis and signaling via the AMPK and PI3kAkt pathways. Multiple genes involved in folliculogenesis including Bmp15 and Igfr1 were also increased, highlighting the dysregulation in oocyte maturation that is seen in PCOS. Interestingly, KEGG analysis also highlighted pathways involved in Parkinson’s disease and depression, the latter of which is well known to be associated with PCOS, but the role of these RNA transcripts in the oocyte with these disorders is unknown at this time (2).

Finally, as these changes in the oocyte mitochondria are transferred to the offspring, this may explain the inheritance pattern seen in the offspring of PCOS patients (53). PCOS is well known to be hereditary, though numerous studies investigating the genetic contributions to the disease have been unable to identify strong gene candidates to explain this pattern (54, 55, 56). Other alternative explanations may include alterations in mitochondrial DNA, increased cellular stress resulting in apoptosis or perhaps via direct effects to the mitochondria itself (26, 29, 42). Furthermore, it stands to reason that the propagation of these mitochondria from a fertilized egg to a blastocyst and ultimately a live-born offspring should result in abnormal mitochondrial function throughout the body. Recent studies in a rat PCOS model have noted mitochondrial dysfunction in the pancreas (57) and kidney (58), and human studies have now reported altered mitochondrial function in skeletal muscle (59) as well as follicular fluid and cumulus cells of PCOS patients (46).

In conclusion, our findings indicate that prenatal androgen programming affects mitochondrial function and structure in oocytes. It is likely that the prenatal administration of androgens during the window of fetal oogenesis affects the mitochondria in the fetal oocyte along with dysregulated transcript abundance.

## Supplementary Material

Supplementary Materials

## Declaration of interest

The authors declare that there is no conflict of interest that could be perceived as prejudicing the impartiality of the research reported.

## Funding

This work was supported by training grants by the Department of Obstetrics and Gynecology, Baylor College of Medicine (N C and B Z) and R-01 research grant (Grant # DK114689) for CSB from National Institutes of Health.
