# Supplementary material for: Prenatal androgen induced lean PCOS impairs mitochondria and mRNA profiles in oocytes
Source: Endocr Connect. 2020 Jan 25;9(3):261–70. doi: 10.1530/EC-19-0553 (PMC7159265; doi:10.1530/EC-19-0553)
Supplement: Supplementary Materials [file supplementary_material.pdf]

Supplementary Materials

Glucose Tolerance Test:

Mice (12 weeks old) in diestrus were fasted for six hours in wire bottom cages. After 6 hours, baseline blood samples for blood glucose were collected from the saphenous vein using the Accucheck Nano Glucometer with Accucheck Smart View Test Strips (Roche, Switzerland). Following baseline measurements, a dose of 1 gram/kilogram glucose (Sigma-Aldrich, MO, USA) was given intra-peritoneally. This was marked as time zero. Further blood samples were obtained at 15, 30, 60, 120 and 180 minutes. Insulin levels were measured with serum collected at each time point in the GTT using a mouse insulin ELISA kit (Mercodia, Sweden) per manufacturer's instructions.

Superovulation and Oocyte Retrieval:

Superovulation was performed using pregnant mare serum gonadotropin (PMSG, Fisher Scientific, Hampton, NH) and human chorionic gonadotropin (hCG, EMD Millipore, Germany). These were both diluted in sterile water and injected intraperitoneally. First, 10 IU of PMSG were injected, followed by 10 IU of hCG approximately 47-52 hours after the PMSG. Mice were euthanized by cervical dislocation under isoflurane anesthesia approximately 18 hours following the hCG injection.

Upon euthanization, the abdominal cavity was opened, and the uterine horns were identified. These were dissected in a cephalad fashion to isolate the distal portion of the uterine horn, the ovary, and the bursa. This section of the reproductive tract was removed and placed in approximately 1000 µl KSOM EmbryoMax media (Sigma, MO, USA) that had been pre-incubated at 37^oC with 5% CO_2 and 6% O_2 conditions. The material was then removed and placed on a pre-incubated petri dish in 200 µl of KSOM. The ampulla was isolated under a microscope and was removed from the remainder of the tissue using sharp dissection and placed in a separate 100 µl droplet of KSOM. Here, gentle traction was applied to the ampulla until a micro-perforation was created, allowing the cumulus oophorous complex (COC) containing the superovulated oocytes

to be released. These were gathered with a 135 µm stripper tip (Origio, Charlottesville, VA) and moved to a clean 100 µl droplet of KSOM. After collection of COCs from both ampullas, they were placed in 0.3 mg/mL hyaluronidase (Sigma, MO, USA) diluted in KSOM for under 30 seconds to denude to cumulus cells and isolate the oocytes. The oocytes were washed 3-5 times in KSOM. All immature oocytes were removed from analysis, and metaphase II (MII) oocytes were isolated and allocated for further study.

Mitochondrial Imaging Studies

Inner Mitochondrial Membrane (IMM) Potential

Oocytes were isolated from each mouse as described above. For IMM, 1 mL of JC-1 dye was mixed daily from stock solution stored at -30°C by diluting 2 µl of stock into 998 µl of PBS, resulting in 2µM concentration of JC-1. Oocytes were incubated in 250 µl of dye for 30 minutes at 37° C, then rinsed in serial PBS washes. The oocytes were then placed on pre-labeled positively charged microscope slides (vWR, Sugarland, TX) in approximately 1 µl of PBS and covered with Prolonged Gold Antifade mounting agent (ThermoFisher, Waltham, MA) and cover slip. Oocytes were immediately visualized using fluorescent microscopy with 20x and 60x objectives using appropriate filters. By comparing the red to green fluorescence ratio, the strength of the IMM was quantified using Image J software.

Reactive Oxygen Species (ROS)

For ROS measurement, 1 mL of CellRox Green dye was mixed daily from stock solution stored at -30° C by diluting 2 µl of stock into 998 µl of PBS. This would result in 5 µM concentration. Oocytes were incubated in 250 µl of dye for 30 minutes at 37° C, then rinsed in serial PBS washes. The oocytes were then placed on pre-labeled positively charged microscope slides (vWR, Sugarland, TX) in approximately 1 µl of PBS and covered with Prolonged Gold Antifade mounting agent (ThermoFisher, Waltham, MA) and cover slip. Oocytes were then immediately visualized using fluorescent microscopy with 20x and 60x objectives and quantified using Image J software.

Lipid Peroxidation

For lipid peroxidation measurement, 1 mL of BODIPY dye was also mixed daily from stock solution stored at -30°C by diluting 1 µl of stock into 999 µl of PBS. After isolation, oocytes were incubated in 4% paraformaldehyde solution (Sigma, MO, USA) for 45 minutes at 37° C. Following this step, oocytes were then placed in 250 µl of BODIPY dye for 1 hour at room temperature, then washed serially in PBS. Oocytes were then placed in approximately 1 µl of PBS on a pre-labeled microscope slide and covered with Prolonged Gold Antifade mounting agent (ThermoFisher, Waltham, MA). The oocytes were immediately visualized with a fluorescent microscope with 20x and 60x objectives. Measurement of fluorescence was performed using Image J software.

ATP Concentration:

For measurement of ATP concentration, 5-15 oocytes from a single mouse were pooled and placed in 45 µl lysis buffer (Promega, Madison, WI, USA), vortexed for 1 minute, then centrifuged at 12,000g for 10 minutes at 4° C. The supernatant was removed and stored in a 200 µl Eppendorf tube at -80° C. Upon collection of all ATP samples, samples were analyzed using an ATP kit utilizing a luciferase assay (ThermoFisher, Waltham, MA) per kit instructions. Standards used for this ELISA ranged from 0, 1, 2.5, 5, 10, 25, 50, and 100 µM, all samples were run in duplicate.

Gene Analysis:

For the collection of DNA and cDNA, three to five oocytes were collected from each mouse and underwent whole genome amplification. For DNA, the Repli-G Single Cell Kit (Qiagen, Germany) was used. The DNA libraries were stored at -80° C until further use. For the creation of cDNA libraries, the WTA Single Cell Kit (Qiagen, USA) was utilized. The cDNA library was stored at -80° C until subsequent use.

Mitochondrial DNA Copy Number

Mitochondrial DNA copy number was assessed using the DNA library created as described above. Real time quantitative PCR (rt-PCR) was performed with 1:500 diluted samples. Reaction mix was prepared fresh daily, and 8 µl was added to each well. Following this, 2 µl of diluted DNA was added. Incubation steps of the protocol were preprogrammed on a thermal cycler. All samples were run in duplicate. For comparison of mitochondrial DNA, a somatic gene (tubulin) was compared with a mitochondrial gene (mtCo1) as previously described [58].

RNA Transcript Abundance

RNA transcript abundance was also measured using real time qPCR, again with diluted samples of cDNA of 1:500. Reaction mix was prepared daily fresh, 8 µl of this mix was added to each well, followed by 2 µl of cDNA sample. Incubation steps of the protocol were preprogrammed on a thermal cycler. Samples were run in duplicate, and GAPDH was used as a housekeeping gene.

Transmission Electron Microscopy:

Electron microscopy imaging was obtained using the contralateral ovary from each mouse described above. The ovaries were placed in a drop of cold primary fix containing 2.5% glutaraldehyde in 0.1M cacodylate buffer to a pH of 7.4 at 4° C. The ovary was then washed in buffer for fifteen minutes 3 times and placed in 1% OsO_4 in 0.1M cacodylate for 45 minutes at 4° C. Following this, the specimens were washed for fifteen minutes 3 times in distilled water, then 50% ethanol twice, 70% ethanol twice, and finally 100% ethanol three times for 20 minutes each. The specimens were then incubated with 2 parts 100% ethanol to one part resin for one hour, then one part 100% ethanol to one part resin overnight, then by one part 100% ethanol to three parts resin for one hour, and finally by pure resin with a catalyst for four hours. The ovaries were then embedded in fresh resin and cured at 62° C for two days. These blocks were used to prepare slides for light microscopy to identify oocytes in the ovarian stroma. Once identified, the tissue block was cut with an ultramicrotome at 50 nanometers and the thin sections were placed on a copper grid and stained with heavy metals to be viewed

under the electron beam of a transmission electron microscope. Images of individual oocytes from each specimen were captured at 2500x, 6000x, 15000x, and 25000x.

Primers Used:

Gene	Gene ID	Sequence ID	Forward 5'-3'	Reverse 5'-3'
Gdf9	14566	NM_008110.2	GGCAGTCTCTTCAGTCCACC	CGGGGAGATCTTTCCACCTC
Bmp15	12155	NM_009757.5	ACAGTGTACCTCAGCCTTCC	ACAGGACTGGGCAATCATACC
Ikbkb	16150	NM_010546.2	TTCCAGCTGAGGAAAGTGTGG	GGCCATGGCGTTCTTCATCTTA
Itga1	109700	NM_001033228.3	CGGAAACGAGAGCTGGCTATT	TCCACAGAGCTAATATAAGCAGCA
Itga4	16401	NM_010576.3	TGCATGAAAGCTGATCAACAC	TGAGGTCTCATCCATTTCCAAGAT
Itga6	16403	NM_008397.4	GACTCTCAACTGCAGCGTCA	GTGCTGTTCCACAACCTGGA
Itgav	16410	NM_008402.3	ACTGCACGGCAGATACAGAG	CACAGCCCAAAGTGTGAACAT
Itgax	16411	NM_021334.2	ACTTTACAAGGCTGGTTTCTTCA	AGTTCTCACTGGGCAACCTG
Pdgfra	18595	NM_011058.3	CCATGCCACCAGTGAAGTCTAT	CGGCAACAGGTTCTCCACTA
Pgr	18667	NM_008829.2	TCCTAAATGAGCAGAGGATGAAG	TCATGGGTCACCTGGAGTTTG
Ppp2ca	19052	NM_019411.4	GCCATGACCGGAACGTAGTA	GCTGGGTCAAACTGCAAGAAAG
Gja1	14609	NM_010288.3	TGATTTCCCTGACGACAGCC	ATCTCCAGGTCATCAGGCCG
Egfr	13649	NM_007912.4	CAACATCCTGGAGGGGGAAC	TGGATGCAGTTGTCTGGTCC
Tuba1b	22143	NM_011654.2	TGGTGTGGATTCTGTGGAAGG	GCCTGCAACTAATACTGGAGC
Tuba1c	22146	NM_009448.4	CTGGTATGTGGGTGAGGGCA	TCGTCTCCTTCAGCACTATCTG
Lhcgr	16867	NM_013582.2	TTGCTGAGCAGATTTGGTTGC	AGCCTGGGAAGGCTTACTTG
Oxtr	18430	NM_001081147.1	CCTGGAGAGACGAGCATTAGC	TCATGCCGAGGATGGTTGAG
Pgrmc1	53328	NM_016783.4	TGGGACTCTCAGTTCACTTTCAA	AGATATGCTCCACCGAATGCT
Plcz1	114875	NM_054066.4	GCCAGAACTGGCATTGATACG	GGAACACGACGATAACCTTTGT
Sfrp4	20379	NM_016687.3	GCCCCCAAAAAGTCAAACCTG	AGTGGGTCCTTAAGGCAAGTG
Zmiz1	328365	NM_001310666.1	TGCAGGAAACTATGCCCCAC	AGGAGCCCCACTGTGATGTA
Amhr2	110542	NM_001356575.1	CCTGTAGGCGACACTGTTCA	AACCCACAGAGAATGACAGCA
Kat2b	18519	NM_001190846.1	CTACAAATGCGCCAGCATCC	ACCTGTTTTAGGCACACTGCT
Xrcc1	22594	NM_009532.4	AACTTTGAGGAGGCCCTGAT	GCTGATGGGGGAGTAACTTCT
Ppp2r1a	51792	NM_016891.3	GCTGCCCACAGTTCTTCGTA	ACTCTGCAGGGTGCTGTTATC
Nobox	18291	NM_130869.3	CCGCACTAGAGGAGGTCAGA	CAACATAGCAGGCCAGTCCA
Igf1	16000	NM_001111274.1	TACTTCAACAAGCCCACAGGC	ATAGAGCGGGCTGCTTTTGT
Igf1r	16001	NM_010513.2	TACAGCGAGGAGAACAAGCC	TGTCTTTCAGGCAGAGGCAG
Ect2	13605	NM_001177626.1	GCACATCATCCTTAGCAGGTA	GTCAGCGTCTTGTTGAAGCA
Eml1	68519	NM_001043335.1	TCACAGTTCCGGGCTCCAAG	GGGACTAAATGACTCGCCACT
Diaph2	54004	NM_172493.2	AATCCAGATAACAGACGGCCC	ATGTCTCCATTCTCCCCGGC
Atrx	22589	NM_009530.2	TGATCAACTGTGTTCAGCGA	GGCTTTGGCATCATGAGGTG
Nbn	27354	NM_013752.3	AGCGCTCTTCAAGAAGATGAA	TGCTGTCCTGAAGACCATCAC
Cep70	68121	NM_023873.3	ATCTTAGAGATTGATGACCTGGATG	CCTTGTCTCAGGATCAGACCTT
Fgfr1-1	14182	NM_001079909.2	TTAGGCAAACCACTTGGCGA	TGCGTCGGACTTCAACATCT
Bmp8a	12163	NM_007558.3	CCATGCCATCTTGCAGTCTC	ACCACCATGTTACGGTGTTT
Nek2	18005	NM_010892.3	GCTAAAGAGCAGGCAGATCCT	CACCACACACAAAGGTTCCC
Nek4	23955	NM_001308328.1	CACTCCCGAGGGAAAAGGAG	ACTCTTCCCCTCCCTGGAATC
Pcm1	18536	NM_023662.3	AGCAGGAGAAGACGTCAGTTT	CCTGGGCAGATGCTTTTCTTG
Spast	50850	NM_016962.2	TGGGTCCTATCCGAGAACTGA	CTGAGGACTCACACTGCGTT
Tacc1	320165	NM_199323.3	CAGATGAAGGTGGAGTCCCTG	CTTTCCCAGCTTTGCGATCA
Opa1	74143	NM_133752.3	ACAAACACTGAAGTTAGGCGA	TTCTTGAGATCTTCTGCCAGC
Dnm1l	74006	NM_152816.3	GATGTGCCAGTTCCAGTTGC	TGCCTTTGGGACACTGTCTT
Mfn1	67414	NM_024200.4	TCCTACTGCTCCTTCTAACCCA	CACTGTTTTCCAAATCACGCCC
Mfn2	170731	NM_001285921.1	GCGCCAGTTTGTGGAATACG	TGTCCCAGACAATTCCTGCT

Gapdh	14433	NM_008084.3	AAGCTCATTTCCTGGTATGACAA	TGGGATAGGGCCTCTCTTGC
Ptgs1	19224	NM_008969.4	GCCCCTATGTTTCCTTCCGT	GGAACAAAACTCCTCCCTCCA
Ptgs2	19225	NM_011198.4	TGGGCCATGGAGTGGACTTA	GGGGATACACCTCTCCACCA
Zp1	22786	NM_009580.2	ACTTGAGCCCTCAGGTTCCA	GCCCAGATCAGACCCACAAA
Zp2	22787	NM_011775.7	GCATCACTGAGGAGCAAACG	TGGGGTCAACACCTTTGGATG
Zp3	22788	NM_011776.1	CCCAGAGTTGGTTGCCAGTA	CGGGGTCCATGGATCTGGAA
mt-ND1	17716	NC_005089.1	TCCGAGCATCTTATCCACGC	GTATGGTGGTACTCCCGCTG
mt-ND2	17717	NC_005089.1	ATCCTCCTGGCCATCGTACT	ATCAGAAGTGGAATGGGGCG
mt-ND3	17718	NC_005089.1	ACAAGCTCTGCACGTCTACC	TGCTCATGGTAGTGGAAGTAGAA
mt-ND4	17719	NC_005089.1	TAATCGCACATGGCCTCACA	CATTTGAAGTCCTCGGGCCA
mt-ND4L	17720	NC_005089.1	CCATACCAATCCCCATCACCA	CGTAATCTGTTCCGTACGTGT
mt-ND5	17721	NC_005089.1	TAGCCTGGCAGACGAACAAG	GTATTCCTGTGAGGGCGAGG
mt-ND6	17722	NC_005089.1	CCGCAAACAAAGATCACCCAG	CTTGATGGTTTGGGAGATTGGT
MT-Cytb	17711	NC_005089.1	TGCATACGCCATTCTACGCT	AGGCTTCGTTGCTTTGAGGT
mt-Co1	17708	NC_005089.1	TCGCAATTCCTACCGGTGTC	CCGGTTAGACCACCAACTGT
mt-Co2	17709	NC_005089.1	CCTGGTGAACTACGACTGCT	GGACTGCTCATGAGTGGAGG
mt-Co3	17710	NC_005089.1	CAAGGCCACCACACTCCTAT	GTCAGCAGCCTCCTAGATCA
mt-ATP6	17705	NC_005089.1	CCAATGGCATTAGCAGTCCG	AATGGTAGCTGTTGGTGGGC
mt-ATP8	17706	NC_005089.1	CAAACATTCCCACTGGCACC	TTGTTGGGGTAATGAATGAGGC
mt-Rnr1	17724	NC_005089.1	TCGGCGTAAAACGTGTCAAC	TTGGGTCTTAGCTGTCGTGT
mt-Rnr2	17725	NC_005089.1	AATTTCGGTTGGGGTGACCT	AGGATTGCGCTGTTATCCCT
